# *F2RL3* Methylation as a Biomarker of Current and Lifetime Smoking Exposures

**DOI:** 10.1289/ehp.1306937

**Published:** 2013-11-22

**Authors:** Yan Zhang, Rongxi Yang, Barbara Burwinkel, Lutz P. Breitling, Hermann Brenner

**Affiliations:** 1 Division of Clinical Epidemiology and Aging Research, and; 2 Molecular Epidemiology, German Cancer Research Center (DKFZ), Heidelberg, Germany; 3 Molecular Biology of Breast Cancer, Department of Obstetrics and Gynecology, University of Heidelberg, Heidelberg, Germany

## Abstract

Background: Recent genome-wide DNA methylation studies have found a pronounced difference in methylation of the *F2RL3* gene (also known as *PAR-4*) in blood DNA according to smoking exposure. Knowledge on the variation of *F2RL3* methylation by various degrees of smoking exposure is still very sparse.

Objectives: We aimed to assess dose–response relationships of current and lifetime active smoking exposure with *F2RL3* methylation.

Methods: In a large population-based study, we quantified blood DNA methylation at *F2RL3* for 3,588 participants using matrix-assisted laser desorption ionization time-of-flight mass spectrometry. Associations of smoking exposure with methylation intensity were examined by multiple linear regression, controlling for potential confounding factors and paying particular attention to dose–response patterns with respect to current and lifetime smoking exposure as well as time since cessation of smoking.

Results: *F2RL3* methylation intensity showed a strong association with smoking status (*p* < 0.0001), which persisted after controlling for potential confounding factors. Clear inverse dose–response relationships with *F2RL3* methylation intensity were seen for both current intensity and lifetime pack-years of smoking. Among former smokers, *F2RL3* methylation intensity increased gradually from levels close to those of current smokers for recent quitters to levels close to never smokers for long-term (> 20 years) quitters.

Conclusions: *F2RL3* methylation is a promising biomarker for both current and long-term past tobacco exposure, and its predictive value for smoking-related diseases warrants further exploration.

Citation: Zhang Y, Yang R, Burwinkel B, Breitling LP, Brenner H. 2014. *F2RL3* methylation as a biomarker of current and lifetime smoking exposures. Environ Health Perspect 122:131–137; http://dx.doi.org/10.1289/ehp.1306937

## Introduction

Tobacco smoking is an established risk factor for a large number of major diseases, including cancer and pulmonary and cardiovascular diseases (Mathers and Loncar 2006; Thun et al. 2010) as well as all-cause mortality (Gellert et al. 2012; Kondo et al. 2011). Ascertainment of smoking exposure in epidemiological studies and in clinical research and practice relies mostly on self-reporting, which is prone to inaccuracy for a variety of reasons, including intentional underreporting and imperfect recall of lifetime exposure. Although a number of biomarkers for current smoking exposure are well established (e.g., cotinine levels in blood, urine, or saliva), biomarkers that reliably reflect duration, intensity, and dynamics of past smoking exposure and which are of obvious relevance for various health outcomes are lacking (Centers for Disease Control and Prevention 2010).

A pronounced difference in blood DNA methylation of the *F2RL3* gene (the coagulation factor II receptor-like 3 gene, also known as *PAR-4*) between heavy smokers and lifelong nonsmokers was recently identified in a hypothesis-free genome-wide study (Breitling et al. 2011) and subsequently verified by genome-wide studies in two additional independent populations (Shenker et al. 2013; Wan et al. 2012). Furthermore, the methylation of *F2RL3* was strongly associated with mortality in a cohort of > 1,000 patients with stable coronary heart disease (Breitling et al. 2012). Taken together, these findings suggest that *F2RL3* methylation may be a highly informative biomarker of the internal effective dose of smoking exposure and which may be highly predictive of adverse smoking effects. However, its association with smoking habits was only discovered very recently, and information on the variation of *F2RL3* methylation by various degrees of active smoking exposure is still very sparse. We therefore aimed to provide a comprehensive analysis of the association of smoking with *F2RL3* methylation in a large population-based sample of older adults, paying particular attention to dose–response patterns with respect to current and lifetime smoking exposure as well as to the length of time since cessation among former smokers.

## Materials and Methods

*Study population*. The study participants were drawn from the baseline population of the ESTHER study [Epidemiologische Studie zu Chancen der Verhütung, Früherkennung und optimierten Therapie chronischer Erkrankungen in der älteren Bevölkerung (Epidemiological Study Assessing Chances of Prevention, Early Detection and Optimized Treatment of Various Chronic Diseases among Older Adults)], a large, population-based cohort study conducted in southwest Germany. Details of the study design have been reported previously (Raum et al. 2007). In brief, 9,949 participants 50–75 years of age (mean age, 62 years) were recruited by their general practitioners during routine health check-ups between July 2000 and December 2002. The study was approved by the ethics committees of the medical faculty of the University of Heidelberg and the medical board of the State of Saarland, Germany. Written informed consent was provided by all participants, and blood was obtained from 9,828 participants (98.8%). Methylation of *F2RL3* was measured in blood DNA among 3,624 participants [those participants who were recruited during the initial 9 months of the enrollment (between July 2000 and March 2001), a representative sample of the overall cohort] on whom the present analysis was based.

*Data collection*. Each participant completed a standardized self-administrated questionnaire that collected information on sociodemographic characteristics, lifestyle factors, medical history, and history of major diseases. In addition, detailed information on lifetime active cigarette smoking was comprehensively ascertained, including age at initiation and intensity at various ages. For former smokers, age at cessation of smoking was also determined. Prevalent diseases such as diabetes or hypertension were identified by medical records from the general practitioners who recruited the study participants. Prevalent cardiovascular disease was defined by either physician-reported coronary heart disease or self-reported history of myocardial infarction, stroke, pulmonary embolism, or revascularisation of coronary arteries. Blood samples were collected, centrifuged, and stored at –80°C until further processing.

*Methylation assessment*. DNA was manually extracted from whole blood samples using a salting out procedure (Miller et al. 1988), through which predominantly leukocyte DNA was obtained. Sequenom matrix-assisted laser desorption ionization time-of-flight (MALDI-TOF) mass spectrometry was used to quantify DNA methylation at a target region within *F2RL3* (Breitling et al. 2011). In brief, DNA samples were first bisulfite converted using the EZ-96 DNA Methylation Gold Kit (Zymo Research, Irvine, CA, USA). Subsequently, polymerase chain reaction (PCR) using the bisulfite-specific primers 5´-agga​aga​gagG​GTTT​ATTA​GTAG​TATG​GTGG​AGGG​-3´ (sense) and 5´-cagt​aata​cgac​tcac​tata​ggga​gaag​gctA​CTTC​TAAA​CTAA​ATAC​CCAC​CAAA-3´ (antisense) (uppercase letters indicate the sequence-specific regions, and the nonspecific tags are shown in lowercase letters) was applied to amplify the target region located in the second exon of *F2RL3* (Breitling et al. 2011), followed by shrimp alkaline phosphatase treatment and RNAse A cleavage (known as T-cleavage) performed according to the manufacturer’s instructions (Sequenom EpiTyper Assay; Sequenom, San Diego, CA, USA). The PCR product fragments were then cleaned by Resin and spotted on 384 SpectroCHIPs by nanodispenser (both from Sequenom). The chips were analyzed by a Bruker Autoflex Mass Spectrometer system (Bruker Biosciences, Billerica, MA, USA) and data were extracted using SpectroACQUIRE, version 3.3.1.3, software and MassARRAY EpiTyper, version 1.0, software (Sequenom). The target region of *F2RL3* contains five CpG sites (hereafter referred to as CpG_1 to CpG_5), and the procedures outlined above allowed quantification of the proportion of 5-methylcytosines (%5mc) at four of the five CpG sites (CpG_2 to CpG_5) because the mass of the cleavage product of CpG_1 was too low to measure using the MassArray. In addition, methylation at CpG_3 showed low test–retest reliability (Pearson correlation coefficient = 0.56) and lower correlations with the other sites (Spearman correlation coefficients of 0.32–0.33, compared with mutual correlations coefficients of ≥ 0.84 between the other three sites), consistent with previous observations (Breitling et al. 2011, 2012); this suggests that methylation at CpG_3 is not well characterized by the MALDI-TOF assay. Therefore, we excluded CpG_3 and included CpG_2, CpG_4, and CpG_5 in the statistical analysis. CpG_2 (Chr 19: 16861552; NCBI build 36.1/hg18) equals cg03636183, the locus identified to be differentially methylated according to smoking exposure by genome-wide studies (Breitling et al. 2011; Shenker et al. 2013; Wan et al. 2012). Because single nucleotide polymorphisms (SNPs) at the primers’ regions or at/near CpGs can influence methylation intensity, the primers were designed excluding SNPs. A search of online databases also did not identify the presence of any SNPs within the target region. Measurements of 96 duplicate samples showed high test–retest reliability and very limited well/position effects [Pearson correlation coefficients for measurable CpGs (CpG_2, CpG_4, and CpG_5) of 0.89–0.91; mean difference ≤ 0.01%5mc]. All the assays were performed by the same operator in the same laboratory. Procedures after bisulfite treatment were processed in batches corresponding to the chips (*n* = 11). Therefore, we included a random effect variable representing the chip in statistical models to control for potential batch effects.

*Statistical analysis*. The study population was first characterized with respect to major sociodemographic characteristics, lifestyle factors, and prevalent diseases. Median and interquartile methylation levels at target CpGs within *F2RL3* were tabulated according to categories defined by these characteristics, and differences were examined by Kruskal–Wallis tests.

Smoking behaviors were classified according to commonly used criteria. An ever-smoker was defined as a subject who had ever smoked ≥ 100 cigarettes during his or her lifetime, thus excluding rare occasional smoking. An ever-smoker was classified as a former smoker if he or she had stopped smoking for ≥ 1 year prior to the study, and as a current smoker otherwise because relapse to smoking mostly occurs within the first year after a quit attempt (Hughes et al. 2004). Cumulative lifetime dose of smoking was assessed by pack-years (a pack-year was defined as having smoked 20 cigarettes per day for 1 year). Intensity of smoking for current smokers was assessed by the average number of cigarettes smoked per day. Median and interquartile methylation levels across categories of the smoking-related variables, including age at initiation, duration, cumulative dose, and current intensity of smoking as well as time since quitting, were calculated separately among current and former smokers, and differences between categories were tested for statistical significance by Kruskal–Wallis tests.

We further examined associations between smoking-related variables and methylation intensity at *F2RL3* using linear regression models, additionally controlling for batch effects and potential confounding factors that were associated with methylation intensity (*p* < 0.05), including age, (years), sex, body mass index [BMI, categorized as underweight (< 18.5 kg/m^2^), normal weight (18.5 to < 25.0 kg/m^2^), overweight (25.0 to < 30.0 kg/m^2^), or obese (≥ 30.0 kg/m^2^)], physical activity [categorized as inactive (< 1 hr/week of physical activity), medium/high (≥ 2 hr/week of vigorous physical activity or ≥ 2 hr/week of light physical activity), or low (all others)], prevalence of cardiovascular disease (yes/no), and diabetes (yes/no). In addition, we performed separate models for current smokers that included both cumulative dose (pack-years) and intensity of smoking (cigarettes per day), and separate models for former smokers that included both cumulative dose and time since smoking cessation. A linear relation between age (modeled as a continuous variable) and methylation intensity was confirmed by modeling age as a restricted cubic spline (Desquilbet and Mariotti 2010). Restricted cubic spline regression was also used to model the shape of dose–response relationships between methylation intensity and smoking-related variables, including intensity of current and lifetime smoking exposure as well as time since cessation of smoking, again controlling for potential confounding factors. Additional analyses by beta-regression designed to model continuous outcome variables with values ranging from 0 to 1 (Ferrari and Cribari-Neto 2004), such as methylation intensities, yielded very similar results; *R*^2^ suggested that goodness of fit was slightly lower than that of linear regression (data not shown). All aforementioned analyses were then repeated using the average methylation intensity at three CpG sites (CpG_2, CpG_4, and CpG_5) as outcomes; the results were consistent with findings for the individual CpGs (data not shown). Because DNA samples were randomly allocated for methylation analysis, characteristics such as age, sex, and smoking categories were equally represented on each plate; consequently, although batch effects were statistically significant, adjusting for batch effects had very little impact on the associations between smoking behaviors and methylation intensity.

All data analyses were conducted using SAS version 9.2 (SAS Institute Inc., Cary, NC, USA). Two-sided *p*-values of < 0.05 were considered statistically significant.

## Results

Of 3,624 participants recruited in the ESTHER study between July 2000 and March 2001, methylation levels at one or more CpG sites could be determined in 3,588 participants (99.0%), who were included in the current analysis. The vast majority of participants (98.2%) were of German nationality. Other characteristics of the study population are shown in Table 1. The sample included more women (56%) than men, and the mean age was 62 years. Approximately 50% of the participants were former or current smokers, > 70% were overweight or obese, > 50% had hypertension, and 17% had cardiovascular disease.

**Table 1:** Baseline characteristics and *F2RL3* (CpG_4) methylation intensity of the study population.

Characteristic	*n* (%)	Methylation intensity	
		Median (Q1–Q3)	*p*‑Value^*a*^
Overall	3,588 (100)	0.79 (0.72–0.84)
Sex
Male	1,594 (44.4)	0.77 (0.66–0.82)
Female	1,994 (55.6)	0.80 (0.75–0.84)	< 0.0001
Age (years)
50–59	1,265 (35.3)	0.79 (0.69–0.84)
60–64	1,025 (28.6)	0.80 (0.72–0.84)
65–69	789 (22.0)	0.79 (0.73–0.84)
70–75	509 (14.2)	0.79 (0.72–0.84)	0.04
BMI (kg/m^2^)^*b*^
Underweight (< 18.5)	21 (0.6)	0.71 (0.62–0.84)
Normal weight (18.5 to < 25.0)	958 (26.8)	0.79 (0.69–0.83)
Overweight (25.0 to < 30.0)	1,692 (47.3)	0.79 (0.73–0.84)
Obesity (≥ 30.0)	908 (25.3)	0.79 (0.72–0.83)	0.005
Smoking status^*c*^
Never smoker	1,701 (48.7)	0.82 (0.78–0.85)
Former smoker	1,136 (32.5)	0.77 (0.70–0.82)
Current smoker	654 (18.7)	0.62 (0.53–0.73)	< 0.0001
Alcohol consumption (g/day)^*d*^
Abstainer	1,052 (32.3)	0.79 (0.71–0.84)
Low (women, 0–19.99; men, 0–39.99)	1,963 (60.2)	0.79 (0.72–0.84)
Intermediate (women, 20–39.99; men, 40–59.99)	191 (5.9)	0.79 (0.71–0.84)
High (women, ≥ 40; men, ≥ 60)	53 (1.6)	0.79 (0.73–0.83)	0.96
Physical activity^*e*^
Inactive	725 (20.3)	0.79 (0.71–0.83)
Insufficient	1,655 (46.2)	0.79 (0.71–0.83)
Sufficient	1,199 (33.5)	0.80 (0.74–0.84)	0.0002
Diabetes^*f*^
Not prevalent	3,011 (84.1)	0.79 (0.72–0.84)
Prevalent	571 (15.9)	0.78 (0.69–0.83)	0.05
Hypertension^*g*^
Not prevalent	1,524 (42.5)	0.79 (0.72–0.84)
Prevalent	2,063 (57.5)	0.79 (0.71–0.83)	0.45
Cardiovascular disease^*h*^
Not prevalent	2,984 (83.2)	0.79 (0.72–0.84)
Prevalent	601 (16.8)	0.78 (0.68–0.82)	< 0.0001
Cancer^*i*^
Not prevalent	3,255 (93.4)	0.79 (0.72–0.84)
Prevalent	231 (6.6)	0.78 (0.71–0.83)	0.18
Q, quartile. ^***a***^Kruskal–Wallis test for group differences. ^***b***^Data missing for 9 participants. ^***c***^Data missing for 97 participants. ^***d***^Data missing for 329 participants. ^***e***^Data missing for 9 participants; categories defined as follows: inactive, < 1 hr/week of physical activity; medium/high: ≥ 2 hr/week of vigorous physical activity or ≥ 2 hr/week of light physical activity; low, other. ^***f***^Data missing for 6 participants. ^***g***^Data missing for 1 participant. ^***h***^Data missing for 3 participants. ^***i***^Data missing for 102 participants.			

*Methylation intensities by demographic and behavioral factors*. We present results for methylation intensity at *F2RL3* CpG_4 in the main text because this site was most strongly associated with mortality in our previous study (Breitling et al. 2012). Corresponding results for CpG_2 and CpG_5 are provided in the Supplemental Material. Examples of mass spectrometry results for CpG_2, CpG_4, and CpG_5 in one participant are shown in Supplemental Material, Figure S1.

**Figure 1:**
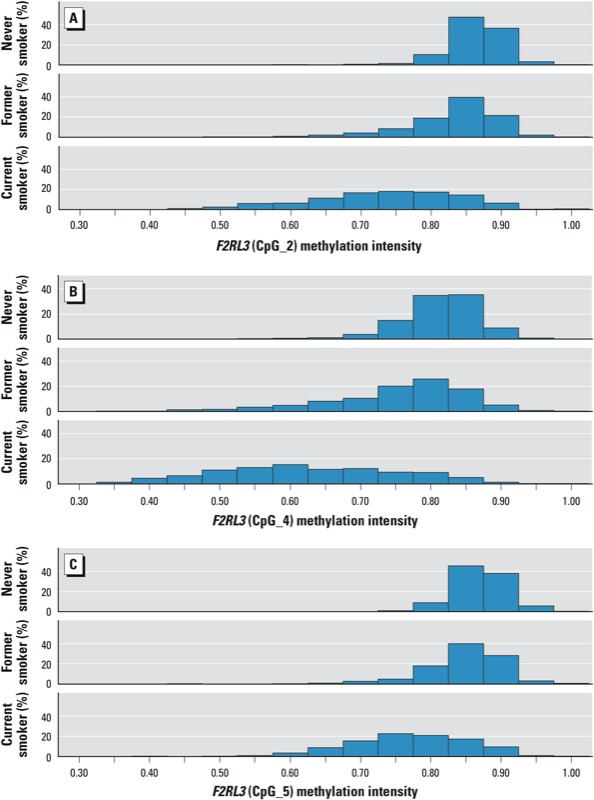
Distribution of *F2RL3* methylation intensity by smoking status (never, former, or current smoker) at *F2RL3* CpG_2 (*A*), *F2RL3* CpG_4 (*B*), and *F2RL3* CpG_5 (*C*). *p* < 0.0001 by Kruskal–Wallis test for each distribution.

Table 1 shows methylation intensities at *F2RL3* CpG_4 across various strata of characteristics of the study population (see Supplemental Material, Table S1, for corresponding results for CpG_2 and CpG_5). Median methylation at all three sites was lower among men than among women, whereas there was very limited variation with respect to age. The small group of underweight participants exhibited lower methylation levels than normal weight, overweight, or obese participants. Compared with participants who never smoked, current and former smokers had the lowest and intermediate methylation levels, respectively. A more comprehensive presentation of the distribution of methylation intensities according to smoking status is shown in Figure 1.

*Methylation intensities by smoking characteristics*. Table 2 shows detailed results on variation of methylation intensities at *F2RL3* CpG_4 according to smoking characteristics among 1,136 former smokers and 654 current smokers (median values for all three loci are reported in Supplemental Material, Table S2). The youngest age for starting tobacco smoking was 10 years. The longest lifetime duration of smoking was up to 60 years for both former and current smokers. The cumulative dose of smoking ranged from 0.5 to 101 and from 0.2 to 147 pack-years for former and current smokers, respectively. The maximum average number of cigarettes smoked per day by current smokers was 60.

**Table 2:** Smoking characteristics and *F2RL3* (CpG_4) methylation intensity of the study population.

Characteristic	Current smokers (*n* = 654)			Former smokers (*n* = 1,136)		
	*n*^*a*^	Methylation intensity [median (Q1–Q3)]	*p*‑Value^*b*^	*n*^*a*^	Methylation intensity [median (Q1–Q3)]	*p*‑Value^*b*^
Age at initiation of smoking (years)^*c*^
10–14	25	0.58 (0.51–0.61)		40	0.76 (0.63–0.81)
15–19	287	0.60 (0.52–0.71)		583	0.77 (0.70–0.82)
20–24	174	0.62 (0.53–0.72)		273	0.78 (0.71–0.82)
25–62	133	0.65 (0.56–0.75)	0.008	174	0.77 (0.67–0.82)	0.18
Lifetime duration of smoking (years)^*d*^
1–19	17	0.68 (0.54–0.75)		107	0.82 (0.78–0.85)
20–29	57	0.64 (0.56–0.72)		300	0.80 (0.76–0.84)
30–39	279	0.62 (0.53–0.72)		320	0.77 (0.71–0.82)
40–60	266	0.61 (0.52–0.70)	0.13	343	0.70 (0.63–0.78)	< 0.0001
Cumulative dose of smoking (pack-years)^*e*^
0.2–9	43	0.72 (0.68–0.80)		243	0.81 (0.77–0.84)
10–19	68	0.69 (0.56–0.76)		256	0.78 (0.74–0.82)
20–29	127	0.62 (0.54–0.71)		208	0.74 (0.67–0.80)
30–147	343	0.59 (0.51–0.68)	< 0.0001	264	0.71 (0.64–0.78)	< 0.0001
Current intensity of smoking (average number of cigarette/day)
1–9	89	0.72 (0.61–0.79)
10–19	153	0.62 (0.52–0.69)
20–29	235	0.60 (0.53–0.69)
30–60	94	0.56 (0.48–0.64)	< 0.0001
Time since cessation of smoking (years)
1				40	0.66 (0.59–0.74)	
2–4				99	0.70 (0.62–0.79)
5–9				145	0.72 (0.65–0.79)
10–19				335	0.76 (0.69–0.82)
20–50				503	0.80 (0.75–0.84)	< 0.0001
Q, quartile. ^***a***^Sum does not always add up to total due to missing values; information on age at initiation and duration of smoking was missing for 66 former smokers and 35 current smokers; information on pack-years was missing for 165 former smokers and 73 current smokers; information on intensity of smoking was missing for 83 current smokers; information on time since cessation of smoking was missing for 14 former smokers. ^***b***^Kruskal–Wallis test for group differences. ­^***c***^Categories for former smokers are: 10–14/15–19/20–24/25–56. ^***d***^Categories for former smokers are 1–9/10–19/20–29/30–60. ^***e***^Categories for former smokers are 0.5–9/10–19/20–29/30–101.						

Among current smokers, strong inverse associations with methylation intensities were seen for both current smoking intensity and lifetime cumulative smoking (Table 2; see also Supplemental Material, Table S2). In addition, young age at smoking initiation was associated with particularly low methylation intensities. Among former smokers, methylation intensities strongly decreased with lifetime duration and cumulative dose of smoking. However, at comparable cumulative doses, methylation intensity was much higher among former smokers than current smokers. Furthermore, methylation intensity was strongly associated with time since smoking cessation. Nevertheless, methylation intensity was close to levels observed in never smokers (median 0.82; IQR 0.78–0.85 for CpG_4) only among former smokers who had quit > 20 years previously (median 0.80; IQR 0.75–0.84).

**Figure 2:**
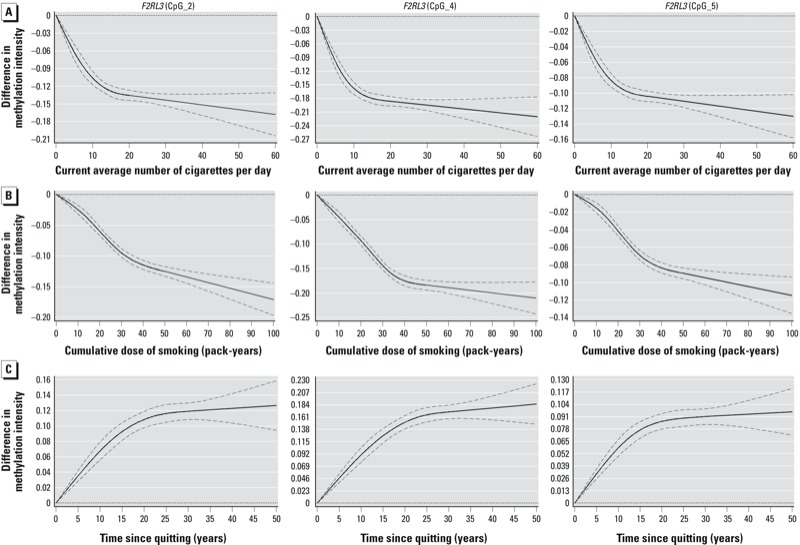
Dose–response relationships between smoking behavior and *F2RL3* methylation intensity (restricted cubic spline regression adjusted for potential confounding factors). (*A*) Dose–response relationship between current intensity of smoking and *F2RL3* methylation intensity (never and former smokers are the reference group, with current smoking intensity = 0). (*B*) Dose–response relationship between cumulative dose of smoking and *F2RL3* methylation intensity (never smokers are the reference group, with pack-years = 0). (*C*) Dose–response relationship between time since cessation of smoking and *F2RL3* methylation intensity among former smokers (current smokers are the reference group, with time since cessation = 0). Dashed lines represent confidence limits.

Table 3 shows the association between smoking behavior and methylation intensities at *F2RL3* CpG_4 estimated by linear regression (corresponding results for CpG_2 and CpG_5 are reported in Supplemental Material, Table S3). Current intensity and cumulative dose of smoking were both inversely associated with methylation intensities, and controlling for potential confounders had very little impact on regression coefficients. Dose–response relationships based on restricted cubic spline models of these factors are shown in Figure 2A,B. We observed a steep decrease in methylation intensities with increasing smoking intensity up to approximately 10–15 cigarettes/day and with a cumulative dose of smoking up to approximately 40 pack-years, with little further decrease at higher current and lifetime smoking exposure (Figure 2A and 2B, respectively). Among former smokers, methylation intensity steadily increased with time since cessation—up to approximately 20–25 years after quitting—and remained essentially stable thereafter (Figure 2C).

**Table 3:** Association between smoking behavior and *F2RL3* (CpG_4) methylation intensity.

Smoking characteristic	Model 1^*a*^		Model 2^*b*^	
	Regression coefficient (95% CI)	*p*‑Value	Regression coefficient (95% CI)	*p*‑Value
Smoking status
Never smoker	Reference		Reference
Former smoker	–0.059 (–0.066, –0.053)	< 0.0001	–0.051 (–0.058, –0.044)	< 0.0001
Current smoker	–0.185 (–0.193, –0.177)	< 0.0001	–0.181 (–0.189, –0.173)	< 0.0001
Current intensity of smoking (average number of cigarettes/day)
0 (never and former smokers)	Reference		Reference
1–9	–0.088 (–0.107, –0.069)	< 0.0001	–0.093 (–0.111, –0.074)	< 0.0001
10–19	–0.181 (–0.196, –0.166)	< 0.0001	–0.178 (–0.192, –0.164)	< 0.0001
20–29	–0.179 (–0.191, –0.167)	< 0.0001	–0.177 (–0.189, –0.166)	< 0.0001
30–60	–0.218 (–0.237, –0.200)	< 0.0001	–0.210 (–0.228, –0.192)	< 0.0001
Cumulative dose of smoking (pack-years)
0 (never smokers)	Reference		Reference
0.2–9	–0.024 (–0.035, –0.013)	< 0.0001	–0.025 (–0.036, –0.014)	< 0.0001
10–19	–0.067 (–0.078, –0.057)	< 0.0001	–0.067 (–0.078, –0.057)	< 0.0001
20–29	–0.123 (–0.134, –0.113)	< 0.0001	–0.123 (–0.133, –0.113)	< 0.0001
30–147	–0.169 (–0.178, –0.161)	< 0.0001	–0.171 (–0.179, –0.162)	< 0.0001
Time since cessation of smoking (years)
0 (current smokers)	Reference		Reference
1	0.022 (–0.006, 0.050)	0.12	0.019 (–0.007, 0.046)	0.16
2–4	0.068 (0.049, 0.086)	< 0.0001	0.071 (0.053, 0.088)	< 0.0001
5–9	0.074 (0.058, 0.090)	< 0.0001	0.079 (0.064, 0.094)	< 0.0001
10–19	0.120 (0.108, 0.131)	< 0.0001	0.121 (0.111, 0.132)	< 0.0001
20–50	0.163 (0.152, 0.173)	< 0.0001	0.171 (0.161, 0.181)	< 0.0001
^***a***^Linear regression without adjustment. ^***b***^Linear regression, adjusted for sex, age, BMI (underweight/normal weight/overweight/obesity), physical activity (inactive/low/medium and high), prevalence of cardiovascular disease and ­diabetes, and batch effect.				

Mutual adjustment for current smoking intensity and cumulative dose among current smokers attenuated associations of methylation intensity with these two factors to a similar degree (Table 4; see also Supplemental Material, Table S4). Among former smokers, mutual adjustment attenuated associations with cumulative dose but had little influence on positive associations between time since quitting and methylation intensities (Table 5; see also Supplemental Material, Table S5).

**Table 4:** Association between smoking behaviors and *F2RL3* (CpG_4) methylation intensity among current smokers (*n* = 654).

Smoking characteristic	Model 1^*a*^		Model 2^*b*^		Model 3^*c*^	
	Regression coefficient (95% CI)	*p*‑Value	Regression coefficient (95% CI)	*p*‑Value	Regression coefficient (95% CI)	*p*‑Value
Cumulative dose of smoking (pack-years)
0.2–9	Reference		Reference		Reference
10–19	–0.056 (–0.102, –0.010)	0.01	–0.067 (–0.111, –0.023)	0.0028	–0.068 (–0.114, –0.023)	0.0036
20–29	–0.095 (–0.137, –0.053)	< 0.0001	–0.104 (–0.144, –0.064)	< 0.0001	–0.092 (–0.135, –0.049)	< 0.0001
30–147	–0.121 (–0.160, –0.083)	< 0.0001	–0.129 (–0.166, –0.091)	< 0.0001	–0.104 (–0.147, –0.060)	< 0.0001
Intensity of smoking (average number of cigarettes/day)
1–9	Reference		Reference		Reference
10–19	–0.093 (–0.125, –0.062)	< 0.0001	–0.081 (–0.111, –0.051)	< 0.0001	–0.054 (–0.086, –0.021)	0.0012
20–29	–0.091 (–0.120, –0.061)	< 0.0001	–0.085 (–0.113, –0.057)	< 0.0001	–0.045 (–0.079, –0.012)	0.0083
30–60	–0.130 (–0.165, –0.095)	< 0.0001	–0.118 (–0.152, –0.084)	< 0.0001	–0.075 (–0.115, –0.035)	0.0003
^***a***^Linear regression without adjustment. ^***b***^Linear regression adjusted for sex, age, BMI (underweight/normal weight/overweight/obesity), physical activity (inactive/low/medium and high), prevalence of cardiovascular disease and diabetes, and batch effect. ^***c***^Linear regression as in model 2, also adjusted for cumulative dose and intensity of smoking.						

**Table 5:** Association between smoking behaviors and *F2RL3* (CpG_4) methylation intensity among former smokers (*n* = 1,136).

Smoking characteristic	Model 1^*a*^		Model 2^*b*^		Model 3^*c*^	
	Regression coefficient (95% CI)	*p*‑Value	Regression coefficient (95% CI)	*p*‑Value	Regression coefficient (95% CI)	*p*‑Value
Cumulative dose of smoking (pack-years)
0.5–9	Reference		Reference		Reference
10–19	–0.034 (–0.050, –0.017)	< 0.0001	–0.031 (–0.047, –0.015)	0.0002	–0.017 (–0.032, –0.001)	0.03
20–29	–0.071 (–0.089, –0.054)	< 0.0001	–0.072 (–0.089, –0.055)	< 0.0001	–0.042 (–0.059, –0.024)	< 0.0001
30–101	–0.099 (–0.115, –0.082)	< 0.0001	–0.095 (–0.112, –0.079)	< 0.0001	–0.044 (–0.062, –0.025)	< 0.0001
Time since cessation of smoking (years)
1	Reference		Reference		Reference
2–4	0.045 (0.011, 0.080)	0.0098	0.056 (0.023, 0.088)	0.0008	0.051 (0.018, 0.084)	0.0027
5–9	0.052 (0.019, 0.084)	0.0020	0.063 (0.032, 0.093)	< 0.0001	0.058 (0.026, 0.089)	0.0003
10–19	0.098 (0.067, 0.128)	< 0.0001	0.104 (0.076, 0.133)	< 0.0001	0.090 (0.061, 0.120)	< 0.0001
20–50	0.140 (0.110, 0.170)	< 0.0001	0.157 (0.129, 0.186)	< 0.0001	0.132 (0.101, 0.163)	< 0.0001
^***a***^Linear regression without adjustment. ^***b***^Linear regression adjusted for sex, age, BMI (underweight/normal weight/overweight/obesity), physical activity (inactive/low/medium and high), prevalence of cardiovascular disease and diabetes, and batch effect. ^***c***^Linear regression as in model 2, also adjusted for cumulative dose and time since cessation of smoking.						

## Discussion

This large population-based study corroborates and expands on recent evidence from several smaller studies that reported a strong association between smoking and *F2RL3* methylation (Breitling et al. 2011; Shenker et al. 2013; Wan et al. 2012). In particular, we found substantially reduced *F2RL3* methylation intensities among smokers (median methylation intensities at CpG_4 among current and former smokers were 0.62 and 0.77, respectively, compared with 0.82 among never smokers), and monotonic dose–response relationships of both current smoking intensity and lifetime amount of smoking with *F2RL3* methylation. Among former smokers, methylation levels increased with time since cessation, but full recovery to levels of nonsmokers was seen only after cessation for > 20 years.

To our knowledge, this is the first study providing detailed dose–response analyses on the association of various indicators of smoking exposure with *F2RL3* methylation. The observed dose–response pattern for current and lifetime exposure closely parallels dose–response patterns seen between smoking and a variety of diseases, including cardiovascular disease and various forms of cancer (Doll et al. 2005; Jacobs et al. 1999; Peto et al. 2000; Teo et al. 2006). Analogies likewise exist regarding dose–response patterns with time since cessation. Although risk of cardiovascular disease tends to approach the lower risk of nonsmokers within relatively short periods of time after cessation (Dobson et al. 1991; Gordon et al. 1974; Kramer et al. 2006; Lightwood and Glantz 1997), reduction of excess risk for cancer typically takes two to three decades (Ebbert et al. 2003; Hrubec and McLaughlin 2007).

The *F2RL3* gene encodes for the thrombin protease-activated receptor-4 (*PAR-4*), which is expressed on the surface of various body tissues, including circulating leukocytes (Vergnolle et al. 2002; Xu et al. 1998). The activation of *PAR-4* has been implicated to be responsible for leukocyte recruitment, modulation of rolling and adherence of leukocytes, such as neutrophils and eosinophils, as well as regulation of vascular endothelial cell activity (Gomides et al. 2012; Kataoka et al. 2003; Leger et al. 2006; Vergnolle et al. 2002). These pathophysiological events are considered to be the early steps of inflammatory reactions in the vascular system (Leger et al. 2006; Steinhoff et al. 2005; Vergnolle et al. 2002) and have also been described in smoking-induced adverse effects (Leone 2007; Rahman and Laher 2007). The expression of DNA methyltransferase-1 (DNMT-1), a key enzyme involved in maintaining methylation (Bhutani et al. 2011), was down-regulated in epithelial cells exposed to cigarette smoke condensate *in vitro* (Liu et al. 2010) and in GABAergic neurons (neurons that produce γ-aminobutyric acid) following nicotine exposure in mice (Satta et al. 2008). In addition, *F2RL3* expression increased as duration of exposure to cigarette smoke increased from 3 to 28 days in mice (*n* = 5), although the changes were not statistically different from controls (Shenker et al. 2013). These findings suggest that a causal relationship between smoking, *F2RL3* methylation, and smoking-associated cardiovascular diseases is plausible. This suggestion is further supported by recent evidence that *F2RL3* methylation was strongly associated with mortality in a cohort of 1,206 patients with stable coronary heart disease [hazard ratios (95% CI) for death from any cause, cardiovascular, and non-cardiovascular diseases were 3.19 (1.64–6.21), 2.32 (0.97–5.58), and 5.16 (1.81–14.7), respectively, for patients in the lowest quartile of methylation at *F2RL3* CpG_4 compared with the highest quartile]. (Breitling et al. 2012). Moreover, *PAR-4* is a thrombin receptor that is involved in blood coagulation (Leger et al. 2006; Macfarlane et al. 2001). Given that up to 90% of cancer patients are characterized by a thrombin-associated hypercoagulable state (Falanga 2005; Gouin-Thibault and Samama 1999), and that the overexpression of PAR4 has been reported in prostate cancer tissue (Black et al. 2007) and in *in vitro* colon cancer cells (Gratio et al. 2009), and is involved in the migration of hepatocellular carcinoma cells (Kaufmann et al. 2007) and chondrosarcoma cells *in vitro* (Chen et al. 2010), smoking-induced hypomethylation at *F2RL3* appears to be a plausible explanation for the up-regulated expression of *PAR-4* observed in cancer pathology. However, the clinical relevance of the smoking-associated hypomethylation of *F2RL3,* and the extent to which the hypomethylation might be involved in mediating the detrimental health effects of smoking, is still uncertain at this time.

Regardless of whether *F2RL3* methylation plays a causal role in smoking-related diseases, it appears to have considerable promise as a marker of cumulative exposure to tobacco smoking. A number of biomarkers for current smoking have been identified and are used to a varying extent in epidemiological studies and clinical practice [e.g., exhaled carbon monoxide, cotinine levels in blood, urine, or saliva, and DNA adducts in target or surrogate tissues (Centers for Disease Control and Prevention 2010)]. However, there is still a lack of biomarkers for long-term past exposure, in particular for lifetime exposure because the biomarkers available to date are mostly characterized by short half-lives. For example, cotinine levels reflect only recent exposure and will return to normal values within 2–7 days after cessation (Society for Research on Nicotine and Tobacco Subcommittee on Biochemical Verification 2002). Similar limitations apply to DNA adducts [e.g., aromatic-DNA adducts with half-lives of 10–12 weeks (Godschalk et al. 2003)], which are commonly used as biomarkers of biological effective dose of carcinogen intake (Lodovici and Bigagli 2009). *F2RL3* methylation may, therefore, be particularly useful as a marker of biologically effective dose reflecting lifetime exposure to smoking, which is often not available in detail and may suffer from recall bias or intentional misreporting in epidemiological and clinical studies and clinical practice. Moreover, even if *F2RL3* methylation is not a direct causal intermediate between smoking and disease, it may serve as an accurate marker of cumulative internal dose and, consequently, smoking-associated disease risk.

Our study has specific strengths and limitations. Strengths include the large sample of participants for whom detailed information on lifetime smoking history and a wide range of covariates was available. Limitations include the cross-sectional design, which precluded direct observations of changes of *F2RL3* methylation over time according to smoking habits. Because of the restricted age range of our study population (50–75 years) and because most smokers started smoking before 30 years of age, it was not possible to assess dose–response relationships between duration of smoking and *F2RL3* methylation during the initial years of smoking. Smoking exposure was self-reported and some misclassification may have occurred due to intentional underreporting or imperfect recall of lifetime history. We measured methylation intensities in DNA extracted from all types of peripheral blood leukocytes. It is well known that methylation intensity may strongly vary between cell types (Adalsteinsson et al. 2012; Wu et al. 2011); therefore, we cannot exclude the possibility that differences in methylation observed in our study might reflect differential distribution of various types of leukocytes. However, the composition of leukocytes does not appear to be affected by smoking to a relevant extent. In a large epidemiological study, the proportions of granulocytes, lymphocytes, and monocytes were 61.3%, 31.4%, and 7.4%, respectively, among current smokers, compared to 60.8%, 31.4%, and 8.0%, respectively, among nonsmokers (Smith et al. 2003). Nevertheless, the potential for confounding to variation in white blood cell subtypes should be addressed in future research, even though such confounding would not diminish the value of *F2RL3* methylation as smoking exposure. Finally, although we controlled for a variety of potential confounding variables, we cannot exclude the possibility that the relationship between smoking and *F2RL3* methylation is explained to some extent by uncontrolled or incompletely controlled confounding variables.

## Conclusions

Despite its limitations, our study strongly suggests that *F2RL3* methylation may be a highly informative biomarker of both current and lifetime smoking exposure. Further research should use longitudinal approaches to clarify the full potential of *F2RL3* methylation as a dynamic summary measurement that may reflect accumulated smoking-associated disease risks better than any other marker available to date.

## Supplemental Material

(393 KB) PDF
