# Supplementary material for: F2RL3 Methylation as a Biomarker of Current and Lifetime Smoking Exposures
Source: Environ Health Perspect. 2013 Nov 22;122(2):131–7. doi: 10.1289/ehp.1306937 (PMC3915264; doi:10.1289/ehp.1306937)
Supplement: (393 KB) PDF [file ehp.1306937.s001.pdf]

Environmental Health Perspectives doi:10.1289/ehp.1306937

Supplemental Material

F2RL3 Methylation as a Biomarker of Current and Lifetime Smoking Exposures

Yan Zhang, Rongxi Yang, Barbara Burwinkel, Lutz P. Breitling, and Hermann Brenner

Table of Contents

Table S1, pages 2-3

Table S2, page 4

Table S3, page 5

Table S4, page 6

Table S5, page 7

Figure S1, page 8

Supplemental Material, Table S1. Baseline characteristics and *F2RL3* methylation intensity of the study population

Characteristics	*F2RL3* CpG2 Median(Q1-Q3)	*P-value*[a]	*F2RL3* CpG4 Median(Q1-Q3)	*P-value*[a]	*F2RL3* CpG5 Median(Q1-Q3)	*P-value*[a]
Overall	0.85 (0.81 – 0.88)		0.79(0.72 – 0.84)		0.86 (0.82 – 0.88)	
Sex						
Male	0.84 (0.77 – 0.87)		0.77(0.66 – 0.82)		0.85 (0.80 – 0.88)	
Female	0.86 (0.82 – 0.88)	<0.0001	0.80(0.75 – 0.84)	<0.0001	0.86 (0.83 – 0.89)	<0.0001
Age (years)						
50-59	0.85 (0.79 – 0.88)		0.79(0.69 – 0.84)		0.86 (0.81 – 0.89)	
60-64	0.85 (0.81 – 0.88)		0.80(0.72 – 0.84)		0.86 (0.82 – 0.89)	
65-69	0.85 (0.82 – 0.88)		0.79(0.73 – 0.84)		0.86 (0.82 – 0.89)	
70-75	0.85 (0.81 – 0.87)	0.04	0.79(0.72 – 0.84)	0.04	0.85 (0.82 – 0.88)	0.06
Body mass index (kg/m^2)						
Underweight (<18.5)	0.81 (0.73 – 0.87)		0.71(0.62 – 0.84)		0.84 (0.79 – 0.88)	
Normal weight (18.5-<25.0)	0.85 (0.79 – 0.88)		0.79(0.69 – 0.83)		0.85 (0.81 – 0.88)	
Overweight (25.0-<30.0)	0.85 (0.81 – 0.88)		0.79(0.73 – 0.84)		0.86 (0.82 – 0.89)	
Obesity (≥30.0)	0.85 (0.81 – 0.88)	0.004	0.79(0.72 – 0.83)	0.005	0.86 (0.82 – 0.88)	0.03
Smoking status						
Never smoker	0.87 (0.84 – 0.89)		0.82(0.78 – 0.85)		0.87 (0.85 – 0.89)	
Former smoker	0.84 (0.80 – 0.87)		0.77(0.70 – 0.82)		0.86 (0.82 – 0.88)	
Current smoker	0.75 (0.67 – 0.81)	<0.0001	0.62(0.53 – 0.73)	<0.0001	0.77 (0.72 – 0.84)	<0.0001
Alcohol consumption (g/d)						
Abstainer	0.85 (0.80 – 0.88)		0.79(0.71 – 0.84)		0.86 (0.82 – 0.89)	
Low	0.85 (0.81 – 0.88)		0.79(0.72 – 0.84)		0.86 (0.82 – 0.89)	
Intermediate	0.85 (0.79 – 0.88)		0.79(0.71 – 0.84)		0.86 (0.82 – 0.89)	
High	0.85 (0.81 – 0.88)	0.86	0.79 (0.73 – 0.83)	0.96	0.87 (0.82 – 0.89)	0.90
Physical activity [b]						
Inactive	0.85 (0.80 – 0.88)		0.79(0.71 – 0.83)		0.85 (0.81 – 0.88)	
Insufficient	0.85 (0.80 – 0.88)		0.79 (0.71 – 0.83)		0.86 (0.82 – 0.88)	
Sufficient	0.85 (0.82 – 0.88)	0.007	0.80(0.74 – 0.84)	0.0002	0.86 (0.83 – 0.89)	0.0006
Diabetes						
Not prevalent	0.85 (0.81 – 0.88)		0.79(0.72 – 0.84)		0.86 (0.82 – 0.89)	
Prevalent	0.85 (0.80 – 0.88)	0.06	0.78(0.69 – 0.83)	0.05	0.85 (0.81 – 0.88)	0.005
Hypertension						
Not prevalent	0.85 (0.81 – 0.88)		0.79(0.72 – 0.84)		0.86 (0.82 – 0.89)	
Prevalent	0.85 (0.80 – 0.88)	0.47	0.79(0.71 – 0.83)	0.45	0.86 (0.82 – 0.88)	0.23

Characteristics	*F2RL3* CpG2 Median(Q1-Q3)	*P-value*[a]	*F2RL3* CpG4 Median(Q1-Q3)	*P-value*[a]	*F2RL3* CpG5 Median(Q1-Q3)	*P-value*[a]
Cardiovascular disease						
Not prevalent	0.85 (0.81 – 0.88)		0.79(0.72 – 0.84)		0.86 (0.82 – 0.89)	
Prevalent	0.85 (0.79 – 0.87)	0.004	0.78(0.68 – 0.82)	<0.0001	0.85 (0.81 – 0.88)	0.003
Cancer						
Not prevalent	0.85 (0.81 – 0.88)		0.79(0.72 – 0.84)		0.86 (0.82 – 0.88)	
Prevalent	0.85 (0.81 – 0.87)	0.30	0.78(0.71 – 0.83)	0.18	0.85 (0.82 – 0.88)	0.36

Abbreviations: Q1, 1st quartile; Q3, 3rd quartile. [a] Kruskal-Wallis test for group differences. [b] Categories defined as follows: inactive, <1 hour/week of physical activity; medium/high: ≥2 hour/week of vigorous physical activity or ≥2 hours/week of light physical activity; low, other.

Supplemental Material, Table S2. Median *F2RL3* methylation intensity of the study population according to smoking characteristics

		Current smokers (n=654)						Former smokers (n=1136)					
		CpG2	*P-value* [a]	CpG4	*P-value* [a]	CpG5	*P-value* [a]	CpG2	*P-value* [a]	CpG4	*P-value* [a]	CpG5	*P-value* [a]
Age at initiation of smoking (years)[b]													
	10-14	0.71		0.58		0.74		0.82		0.76		0.83	
	15-19	0.72		0.60		0.76		0.84		0.77		0.86	
	20-24	0.74		0.62		0.77		0.85		0.78		0.86	
	25-62	0.77	0.002	0.65	0.008	0.80	0.005	0.84	0.16	0.77	0.18	0.85	0.03
Lifetime duration of smoking (years)[c]													
	1-19	0.78		0.68		0.81		0.87		0.82		0.87	
	20-29	0.77		0.64		0.78		0.86		0.80		0.87	
	30-39	0.74		0.62		0.77		0.84		0.77		0.85	
	40-59	0.73	0.11	0.61	0.13	0.77	0.16	0.81	<0.0001	0.70	<0.0001	0.83	<0.0001
Cumulative dose of smoking (pack-years)[d]													
	0.2- 9	0.82		0.72		0.84		0.86		0.81		0.87	
	10-19	0.80		0.69		0.81		0.85		0.78		0.86	
	20-29	0.74		0.62		0.77		0.83		0.74		0.84	
	30-147	0.72	<0.0001	0.59	<0.0001	0.76	<0.0001	0.81	<0.0001	0.71	<0.0001	0.84	<0.0001
Current intensity of smoking (average number of cigarette /day)													
	1- 9	0.81		0.72		0.82							
	10-19	0.74		0.62		0.77							
	20-29	0.73		0.60		0.76							
	30-60	0.68	<0.0001	0.56	<0.0001	0.74	<0.0001						
Time since cessation of smoking (years)													
	1							0.78		0.66		0.82	
	2-4							0.81		0.70		0.83	
	5-9							0.81		0.72		0.83	
	10-20							0.84		0.76		0.86	
	20-50							0.86	<0.0001	0.80	<0.0001	0.87	<0.0001

[a] Kruskal-Wallis test for group differences. [b] Categories for former smokers are: 10-14/15-19/20-24/25-56. [c] Categories for former smokers are: 1-9/10-19/20-29/30-60. [d] Categories for former smokers are: 0.5-9/10-19/20-29/30-101.

Supplemental Material, Table S3. Association between smoking behavior and *F2RL3* methylation intensity

Smoking characteristics	*F2RL3* CpG2 Regression coefficient [a] (95% CI)	*P-value*	*F2RL3* CpG4 Regression coefficient [a] (95% CI)	*P-value*	*F2RL3* CpG5 Regression coefficient [a] (95% CI)	*P-value*
Smoking status						
Never smoker	Ref.		Ref.		Ref.	
Former smoker	-0.030 (-0.036, -0.024)	<0.0001	-0.051 (-0.058, -0.044)	< 0.0001	-0.017 (-0.022, -0.013)	< 0.0001
Current smoker	-0.125 (-0.132, -0.118)	<0.0001	-0.181 (-0.189, -0.173)	< 0.0001	-0.094 (-0.099, -0.088)	< 0.0001
Current intensity of smoking (average number of cigarettes /day)						
0 (Never and former)	Ref.		Ref.		Ref.	
1-9	-0.058 (-0.073, -0.042)	<0.0001	-0.093 (-0.111, -0.074)	<0.0001	-0.047 (-0.058, -0.035)	<0.0001
10-19	-0.124 (-0.136, -0.112)	<0.0001	-0.178 (-0.192, -0.164)	<0.0001	-0.097 (-0.106, -0.088)	<0.0001
20-29	-0.126 (-0.136, -0.116)	<0.0001	-0.177 (-0.189, -0.166)	<0.0001	-0.100 (-0.107, -0.093)	<0.0001
30-60	-0.162 (-0.177, -0.147)	<0.0001	-0.210 (-0.228, -0.192)	<0.0001	-0.118 (-0.129, -0.106)	<0.0001
Cumulative dose of smoking (pack-years)						
0 (Never smokers)	Ref.		Ref.		Ref.	
0.2-9	-0.016 (-0.025, -0.006)	0.0012	-0.025 (-0.036, -0.014)	<0.0001	-0.008 (-0.016, -0.001)	0.03
10-19	-0.043 (-0.052, -0.034)	<0.0001	-0.067 (-0.078, -0.057)	<0.0001	-0.028 (-0.035, -0.021)	<0.0001
20-29	-0.077 (-0.086, -0.068)	<0.0001	-0.123 (-0.133, -0.113)	<0.0001	-0.057 (-0.064, -0.050)	<0.0001
30-147	-0.117 (-0.125, -0.109)	<0.0001	-0.171 (-0.179, -0.162)	<0.0001	-0.083 (-0.089, -0.077)	<0.0001
Time since cessation of smoking (years)						
0 (Current smokers)	Ref.		Ref.		Ref.	
1	0.020 (-0.004, 0.043)	0.10	0.019 (-0.007, 0.046)	0.16	0.020 (0.003, 0.038)	0.02
2-4	0.059 (0.043, 0.074)	<0.0001	0.071 (0.053, 0.088)	<0.0001	0.051 (0.039, 0.063)	<0.0001
5-9	0.064 (0.051, 0.077)	<0.0001	0.079 (0.064, 0.094)	<0.0001	0.057 (0.047, 0.067)	<0.0001
10-20	0.089 (0.080, 0.099)	<0.0001	0.121 (0.111, 0.132)	<0.0001	0.074 (0.066, 0.081)	<0.0001
20-50	0.122 (0.114, 0.131)	<0.0001	0.171 (0.161, 0.181)	<0.0001	0.096 (0.089, 0.102)	<0.0001

[a] Linear regression, adjusted for sex, age, BMI (underweight/ normal weight/ overweight/ obesity), physical activity (inactive/ low/ medium and high), prevalence of cardiovascular disease and diabetes, and batch effect.

Supplemental Material, Table S4. Associations between smoking behavior and *F2RL3* methylation intensity among current smokers (n=654) after mutual control for smoking exposure variables

Smoking characteristics	*F2RL3* CpG2 Regression coefficient [a] (95% CI)	*P-value*	*F2RL3* CpG4 Regression coefficient [a] (95% CI)	*P-value*	*F2RL3* CpG5 Regression coefficient [a] (95% CI)	*P-value*
Cumulative dose of smoking (pack-years)						
0.2-9	Ref.		Ref.		Ref.	
10-19	-0.042 (-0.084, 0.000)	0.05	-0.068 (-0.114, -0.023)	0.0036	-0.032 (-0.063, -0.001)	0.04
20-29	-0.066 (-0.105, -0.026)	0.0013	-0.092 (-0.135, -0.049)	<0.0001	-0.052 (-0.081, -0.023)	0.0005
30-147	-0.073 (-0.113, -0.033)	0.0003	-0.104 (-0.147, -0.060)	<0.0001	-0.053 (-0.082, -0.024)	0.0004
Intensity of smoking (average number of cigarette /day)						
1-9	Ref.		Ref.		Ref.	
10-19	-0.043 (-0.073, -0.013)	0.0051	-0.054 (-0.086, -0.021)	0.0012	-0.033 (-0.055, -0.011)	0.0028
20-29	-0.038 (-0.068, -0.007)	0.02	-0.045 (-0.079, -0.012)	0.0083	-0.032 (-0.055, -0.009)	0.0055
30-60	-0.069 (-0.106, -0.032)	0.0003	-0.075 (-0.115, -0.035)	0.0003	-0.046 (-0.073, -0.019)	0.0009

[a] Linear regression, adjusted for sex, age, BMI (underweight/ normal weight/ overweight/ obesity), physical activity (inactive/ low/ medium and high), prevalence of cardiovascular disease and diabetes, and batch effect, as well as cumulative dose and current intensity of smoking each other.

Supplemental Material, Table S5. Associations between smoking behavior and *F2RL3* methylation intensity among former smokers (n=1136) after mutual control for smoking exposure variables

Smoking characteristics	*F2RL3* CpG2 Regression coefficient [a] (95% CI)	*P-value*	*F2RL3* CpG4 Regression coefficient [a] (95% CI)	*P-value*	*F2RL3* CpG5 Regression coefficient [a] (95% CI)	*P-value*
Cumulative dose of smoking (pack-years)						
0.5-9	Ref.		Ref.		Ref.	
10-19	-0.008 (-0.021, 0.005)	0.21	-0.017 (-0.032, -0.001)	0.03	-0.006 (-0.016, 0.004)	0.24
20-29	-0.016 (-0.031, -0.001)	0.03	-0.042 (-0.059, -0.024)	<0.0001	-0.014 (-0.025, -0.002)	0.02
30-101	-0.020 (-0.036, -0.005)	0.01	-0.044 (-0.062, -0.025)	<0.0001	-0.010 (-0.022, -0.002)	0.10
Time since cessation of smoking (years)						
1	Ref.		Ref.		Ref.	
2-4	0.038 (0.010, 0.066)	0.0089	0.051 (0.018, 0.084)	0.0027	0.031 (0.009, 0.052)	0.0057
5-9	0.044 (0.017, 0.071)	0.0015	0.058 (0.026, 0.089)	0.0003	0.037 (0.017, 0.058)	0.0004
10-20	0.063 (0.038, 0.088)	<0.0001	0.090 (0.061, 0.120)	<0.0001	0.051 (0.032, 0.070)	<0.0001
20-50	0.095 (0.069, 0.121)	<0.0001	0.132 (0.101, 0.163)	<0.0001	0.072 (0.052, 0.092)	<0.0001

[a] Linear regression, adjusted for sex, age, BMI (underweight/ normal weight/ overweight/ obesity), physical activity (inactive/ low/ medium and high), prevalence of cardiovascular disease and diabetes, and batch effect, as well as cumulative dose and time since cessation of smoking each other.

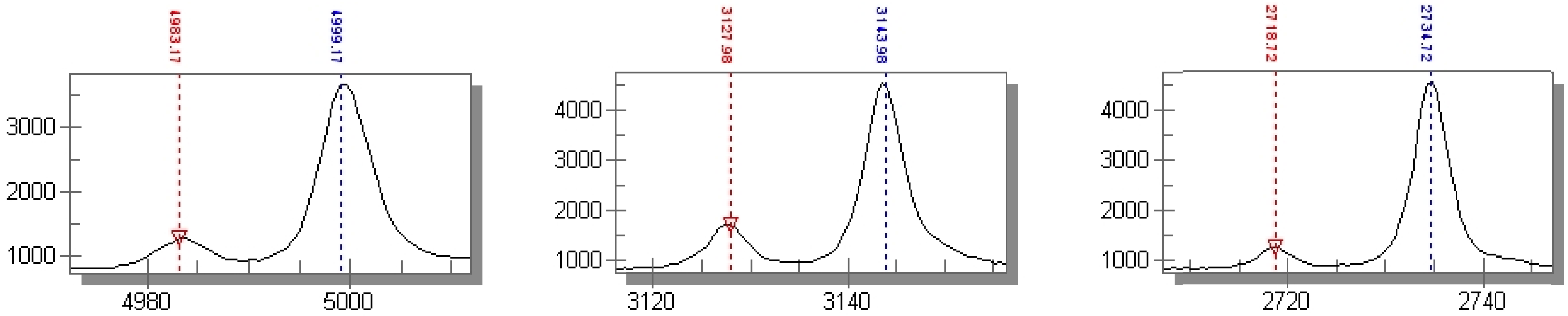

F2RL3 CpG_2 *F2RL3* CpG_4 *F2RL3* CpG_5

Supplemental Material, Figure S1. Examples for results of MassArray Analysis of *F2RL3* methylation. The graphs show the detection intensity (y-axis) of amplicon fragments after base-specific cleavage. Different masses for originally methylated and non-methylated CpG are labeled on the x-axis. The proportion of methylated CpG at these loci thus can be determined by the ratio of detection intensities at masses corresponding to fragments originating from methylated and non-methylated CpG (blue and red vertical lines, respectively). The graphs were taken from a single sample with 86%, 80%, and 90% methylation at *F2RL3* CpG_2, CpG_4 and CpG5, respectively (from left to right).
